# Weight gain as a cause of ineffective drug therapy and failed subcutaneous defibrillator shocks in a paediatric patient with Jervell and Lange-Nielsen syndrome: a case report

**DOI:** 10.1093/ehjcr/ytag341

**Published:** 2026-05-08

**Authors:** Ismael Arco Adamuz, Francesca Perin, Rosa Macías Ruiz, José Manuel Garrido Jimenez, Juan Jiménez-Jáimez

**Affiliations:** Cardiology Department, Virgen de las Nieves University Hospital, Avenida de las Fuerzas Armadas 2, Granada 18014, Spain; Instituto de Investigación Biosanitaria de Granada IBS, Avenida de las Fuerzas Armadas 2, Granada 18014, Spain; Instituto de Investigación Biosanitaria de Granada IBS, Avenida de las Fuerzas Armadas 2, Granada 18014, Spain; Pediatric Department, Virgen de las Nieves University Hospital, Avenida de las Fuerzas Armadas 2, Granada 18014, Spain; Cardiology Department, Virgen de las Nieves University Hospital, Avenida de las Fuerzas Armadas 2, Granada 18014, Spain; Instituto de Investigación Biosanitaria de Granada IBS, Avenida de las Fuerzas Armadas 2, Granada 18014, Spain; Instituto de Investigación Biosanitaria de Granada IBS, Avenida de las Fuerzas Armadas 2, Granada 18014, Spain; Cardiac Surgery Department, Virgen de las Nieves University Hospital, Avenida de las Fuerzas Armadas 2, Granada 18014, Spain; Universidad de Granada, Avenida Doctor Jesús Cadel, Granada 18016, Spain; Cardiology Department, Virgen de las Nieves University Hospital, Avenida de las Fuerzas Armadas 2, Granada 18014, Spain; Instituto de Investigación Biosanitaria de Granada IBS, Avenida de las Fuerzas Armadas 2, Granada 18014, Spain; Universidad de Granada, Avenida Doctor Jesús Cadel, Granada 18016, Spain

**Keywords:** Case report, Jervell and Lange-Nielsen syndrome, Subcutaneous ICD, Paediatric arrhythmia, Shock impedance, Weight gain, Nadolol underdosing, Ineffective ICD therapy

## Abstract

**Background:**

Jervell and Lange-Nielsen syndrome (JLNS) is a rare inherited disorder characterized by congenital bilateral sensorineural deafness and a prolonged QT interval, predisposing to life-threatening arrhythmias. This case illustrates the challenges in the management of a paediatric patient with JLNS, highlighting the impact of weight gain on beta-blocker dosing and subcutaneous implantable cardioverter-defibrillator (S-ICD) efficacy.

**Case summary:**

A 13-year-old male, with a history of severe sensorineural hearing loss, recurrent emotion-triggered seizures, and JLNS diagnosed at age 4, presented with syncope and received three ICD shocks for ventricular fibrillation (VF). His weight gain (from 60 to 79 kg, with a body mass index (BMI) increase from 26.5 to 31.5) resulted in a dose reduction of nadolol to ∼1 mg/kg/day before subsequent dose increase according to body weight gain and failure of the device to deliver effective shocks. Shock impedance was significantly elevated due to increased subcutaneous fat and suboptimal lead positioning. After repositioning, the S-ICD lead to a juxta-sternal position, shock impedance improved (85 Ω), and the patient remained arrhythmia-free after 1 year of follow-up.

**Conclusion:**

Effective management of long qt syndrome (LQTS) requires diligent monitoring of weight, device function, and pharmacological dosing, which is particularly crucial in patients with JLNS. In paediatric patients, rapid weight gain can lead to both underdosing of beta-blockers and mechanical failures of ICD systems. Lifestyle modifications, including regular exercise and a heart-healthy diet, are crucial to prevent obesity and optimize long-term outcomes.

Learning points

- **Jervell and Lange-Nielsen syndrome (JLNS)** is a high-risk congenital arrhythmia syndrome requiring strict pharmacologic and device-based management.
- **Rapid weight gain** in adolescents with subcutaneous implantable cardioverter-defibrillators (ICDs) may lead to **increased shock impedance** and ineffective defibrillation therapy due to adipose tissue accumulation.
- **Underdosing of nadolol** due to unadjusted weight gain can contribute to breakthrough arrhythmias in JLNS patients.
- Close monitoring of **body weight, medication dosing**, and **ICD function** is essential in paediatric patients with inherited arrhythmia syndromes.
- A **multidisciplinary approach**, including lifestyle counselling, nutritional guidance, and regular device evaluation is crucial to optimize outcomes in this vulnerable population.

## Introduction

Jervell and Lange-Nielsen syndrome (JLNS) is a rare and severe form of congenital long QT syndrome (LQTS) characterized by profound sensorineural hearing loss and a high risk of malignant ventricular arrhythmias. Genetically, JLNS can be associated with compound heterozygous mutations affecting both KCNQ1 and KCNE1 genes, which encode subunits of the cardiac potassium channel complex. Homozygous mutations in KCNQ1 have also been described as causative for JLNS.

In paediatric patients, management requires optimal beta-blocker dosing (ideally nadolol at a dose of 2 mg/kg), often combined with an implantable cardioverter-defibrillator (ICD) and/or left cardiac sympathetic denervation (LCSD) as multimodal preventive therapy for sudden cardiac death.^1^ Herein, we report a unique case of ineffective subcutaneous ICD (S-ICD) shocks and subtherapeutic dose nadolol therapy in a paediatric patient with JLNS due to rapid and unnoticed weight gain.

## Summary figure

**Figure:**
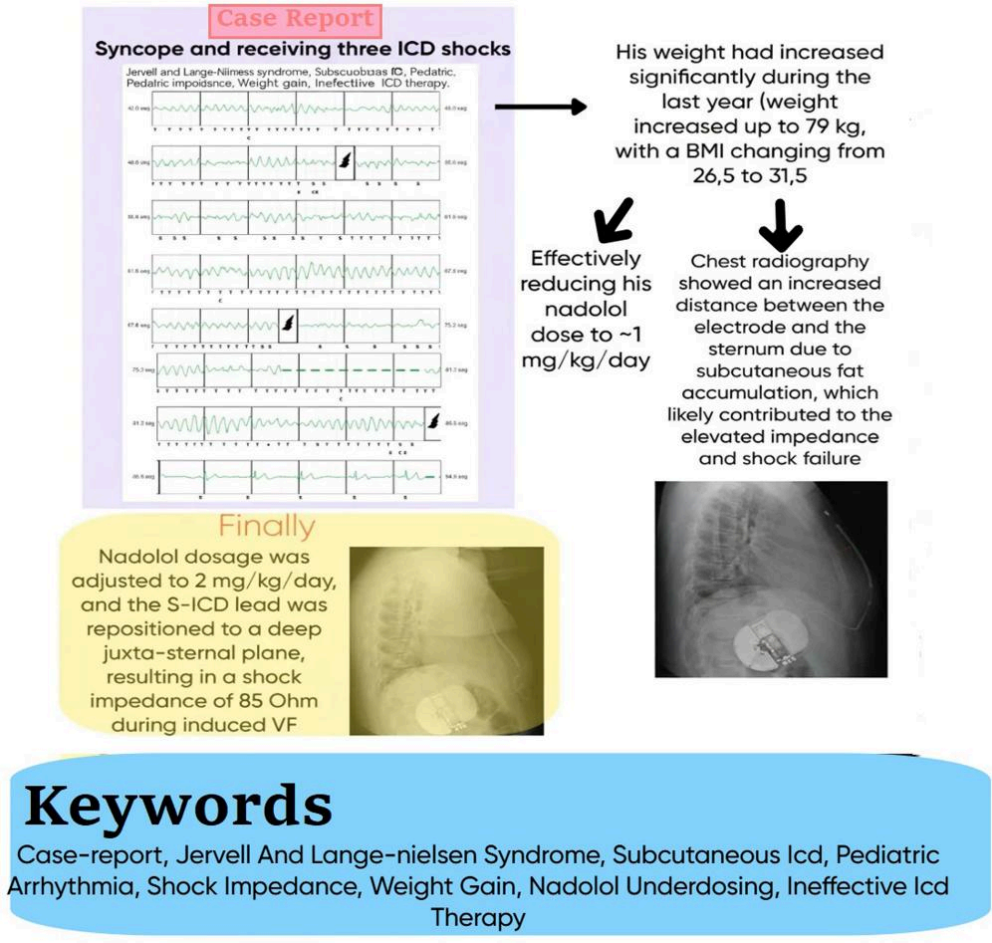


## Case presentation

A 13-year-old male with a history of severe sensorineural hearing loss and recurrent emotion-triggered seizures since infancy had been diagnosed with JLNS at age 4. The diagnosis was based on a corrected QT interval (QTc) of 500 ms and a homozygous deletion in exon 5 of the KCNQ1 gene. Despite the initial propranolol treatment, syncopal episodes persisted, prompting endocardial ICD implantation at the age of 3 years old. The patient later experienced multiple appropriate ICD shocks for ventricular fibrillation (VF), which ceased following left cardiac sympathetic denervation (LCSD). He remained asymptomatic for 6 years under regular outpatient follow-up every 6 months.

Due to a system infection, the endocardial system was explanted and replaced with a S-ICD at 4 years old. During the procedure, VF was induced, and the device appropriately terminated the arrhythmia with the first 65 J shock and a shock impedance of 96 Ω, being the lead placed at the closest possible position to the sternum. At this point, the patient weight was considered normal (60 kg and a body mass index (BMI) of 26.5)

**Figure 1:**
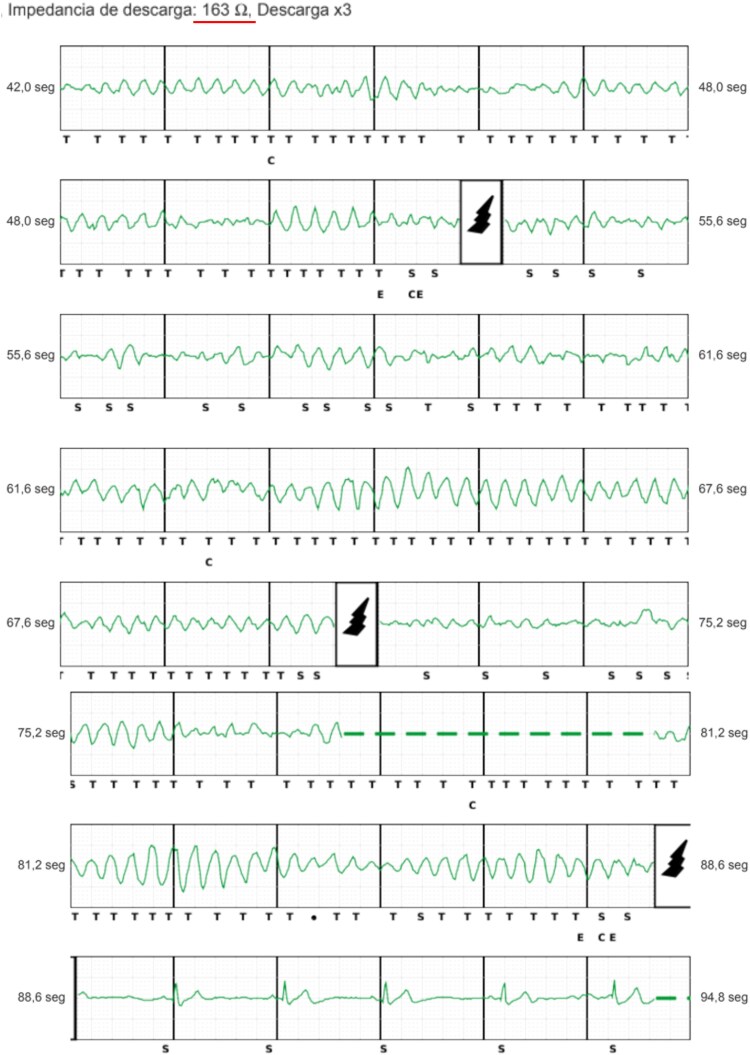
Ineffective shocks from the subcutaneous implantable cardioverter-defibrillator. Appropriately sensed episode of ventricular fibrillation requiring three shocks to restore sinus rhythm (shock impedance 163 Ω).

**Figure 2:**
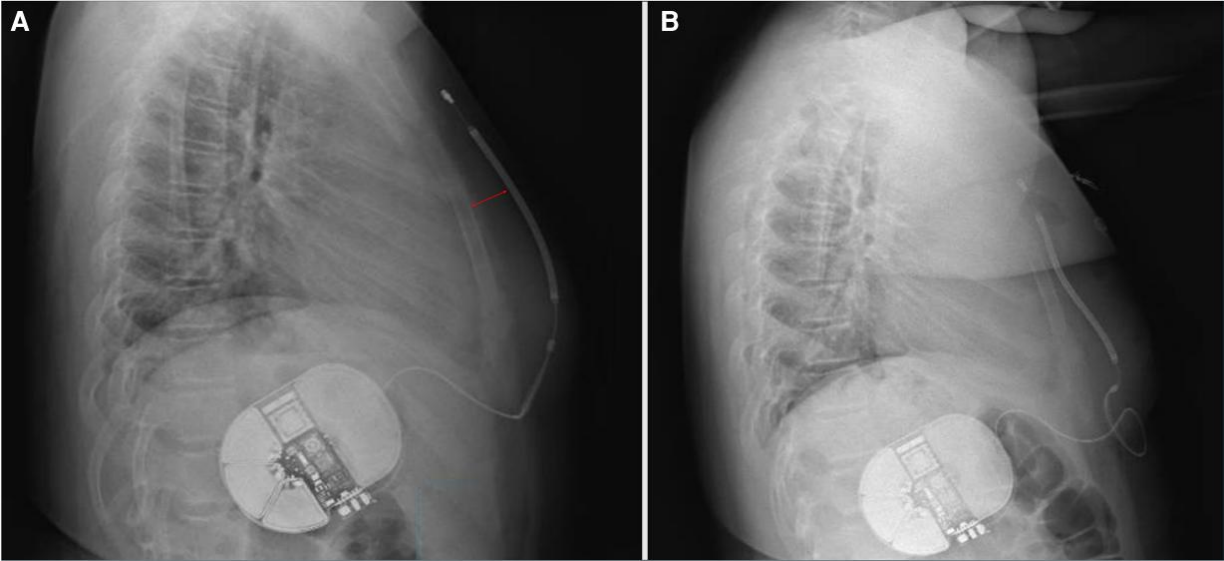
Pre- and post-intervention chest radiographs. (*A*) Increased distance between the electrode and the sternum (arrow) due to subcutaneous fat accumulation, resulting in elevated shock impedance and ineffective therapies. (*B*) Proper electrode positioning after surgical repositioning.

At age 13, the patient was admitted after experiencing syncope and receiving three ICD shocks. He was on nadolol 80 mg daily. Notably, his weight had increased significantly during the last year (weight increased up to 79 kg, with a BMI changing from 26.5 to 31.5, corresponding to an 80% of percentile for height), effectively reducing his nadolol dose to ∼1 mg/kg/day. Device interrogation revealed three VF episodes, the third requiring up to three shocks (at 80 J) to restore sinus rhythm. The first two were ineffective, with a shock impedance of 163 Ω (*Figure 1*). Chest radiography showed an increased distance between the electrode and the sternum due to subcutaneous fat accumulation (*Figure 2A*), which likely contributed to the elevated impedance and shock failure. The praetorian score from the pre-repositioning chest radiograph was estimated at 120 points, indicating intermediate risk with a suboptimal device positioning. Nadolol dosage was adjusted to 2 mg/kg/day, and the S-ICD lead was repositioned to a deep juxta-sternal plane (*Figure 2B*), resulting in a shock impedance of 85 Ω during induced VF. On discharge, the patient and family received counselling on weight management, and follow-up intervals were shortened to allow closer monitoring of weight, device parameters, and pharmacological therapy. After a 1-year follow-up period, the patient has remained free of new arrhythmias and ICD therapies.

## Discussion

JLNS syndrome is associated with high arrhythmic risk, requiring meticulous long-term management.^2^ This case emphasizes the critical importance of maintaining adequate beta-blocker dosing, regular weight monitoring, and ensuring optimal ICD function. Suboptimal dosing due to unnoticed weight gain led to recurrent VF episodes. In adolescents, poor treatment adherence or underdosing due to growth-related changes is common and may result in life-threatening events.^3^ Nadolol is generally preferred in JLNS due to its long half-life and higher proven efficacy to other beta-blockers, with recommended dosing up to 2 mg/kg/day. Failure to adjust beta-blocker dosage during periods of rapid growth or weight gain may result in subtherapeutic exposure and recurrence of malignant arrhythmias. This is particularly important in childhood and adolescence, given the significant physiological changes, including rapid growth and variations in body composition.^4^

Moreover, this case highlights a rarely reported complication of S-ICD therapy: ineffective shocks due to increased shock impedance caused by subcutaneous fat accumulation and a slightly inferior dislodgment of the generator.^5^ In growing adolescents, changes in body composition may significantly increase the distance between the electrode and the myocardium, impairing shock efficacy. In patients with substantial weight gain, reassessment of S-ICD positioning using chest radiography and recalculation of the praetorian score should be considered as a first step. In selected high-risk cases, defibrillation testing under sedation may be warranted to ensure adequate system perfomance.^6^

Given the dynamic physiological changes during adolescence, closer follow-up intervals are recommended, particularly during periods of rapid growth or weight change, to allow timely adjustment of pharmacological therapy and device assessment. Obesity, particularly in paediatric patients with inherited cardiac conditions where physical activity is often restricted,^7^ can adversely affect device performance. Recognizing this mechanism is crucial to prevent treatment failure in similar contexts, given the dynamic changes observed in paediatric and growing patients with evolving body composition. In this regard, an optimal initial lead placement with a target shock impedance below 90 Ω is mandatory.

## Conclusion

In patients with LQTS, and particularly in JLNS, maintaining appropriate beta-blocker dosing and ensuring proper device function are essential. Rapid weight gain can lead to both pharmacologic underdosing and mechanical issues affecting S-ICD efficacy. Weight and lifestyle monitoring are therefore critical. Importantly, recent guidelines support moderate-to-vigorous daily exercise in patients with LQTS,^8^ which, together with a heart-healthy diet, may help prevent obesity and its associated complications.

## Data Availability

The data underlying this article are available in the article.

## Lead author biography

Juan Jiménez Jáimez is a cardiologist specializing in electrophysiology and inherited heart diseases, with 17 years of clinical research experience. He is the Head of the Cardiology Section at Virgen de las Nieves University Hospital and coordinator of the Arrhythmia and Inherited Cardiopathy Unit. He has performed over 1000 ablation and device implantation procedures and has received advanced training in inherited arrhythmic risk cardiomyopathies in London and Chicago. He is the author of more than 100 publications in high-impact journals (*NEJM*, *JACC*, *JAMA*), supervisor of seven doctoral theses, and director of the research group ‘Genetics and Arrhythmias’ of Granada (CTS-1064). He is principal investigator in competitively funded projects and is currently an affiliated professor of medicine at the University of Granada.

## Notes

**Consent:** The authors confirm that written consent for submission and publication of this case series, including images and associated text, has been obtained from the patients, in line with the COPE guidelines. Informed consent was obtained from the patient. Sex and gender variables were considered in accordance with the SAGER guidelines.
